# Comparison of Three Distinct Prophylactic Agents Against Invasive Fungal Infections in Patients Undergoing Haplo-identical Hematopoietic Stem Cell Transplantation and Post-transplant Cyclophosphamide

**DOI:** 10.4084/MJHID.2015.048

**Published:** 2015-08-20

**Authors:** Jean El-Cheikh, Roberto Crocchiolo, Andrea Vai, Sabine Furst, Stefania Bramanti, Barbara Sarina, Angela Granata, Catherine Faucher, Bilal Mohty, Samia Harbi, Reda Bouabdallah, Norbert Vey, Armando Santoro, Christian Chabannon, Luca Castagna, Didier Blaise

**Affiliations:** 1 Unité de Transplantation et de Thérapie Cellulaire (U2T), Institut Paoli-Calmettes, Marseille, France; 2 Departement d’Onco-Hématologie, Institut Paoli-Calmettes, Marseille, France; 3 Centre de Thérapie Cellulaire, Institut Paoli-Calmettes, Marseille, France; 4 Humanitas cancer Centre, Milan, Rozzano, Italy

## Abstract

Over the past decade, invasive fungal infections (IFIs) have remained an important problem in patients undergoing allogeneic haematopoietic stem cell transplantation (Allo-HSCT). The optimal approach for prophylactic antifungal therapy has yet to bedetermined.

We conducted a retrospective analysis, comparing the safety and efficacy of micafungin 50mg/day vs. fluconazole 400mg/day vs. itraconazole 200mg/day as prophylaxis for adult patients with various haematological diseases receiving haploidentical hematopoietic stem cell transplantation (haplo-HSCT) followed by high-dose cyclophosphamide (PT-Cy).

Overall, 99 patients were identified: 30 patients received micafungin, 50 and 19 patients received itraconazole and fluconazole, respectively. After a median follow-up of 12 months (range: 1–51), proven or probable IFIs were reported in 3 patients (10%) in the micafungin, 5 patients in the itraconazole (10%) and 2 patients (11%) in the fluconazole group (p=0.998). Fewer patients in the micafungin group had invasive aspergillosis (1 [3%] vs. 3 [6%] in the itraconazole vs. 2 [11%] in the fluconazole group, p=0.589). Four patients (13%) in the micafungin group vs 13 (26%) patients in the itraconazole group and 10 (53%) patients in the fluconazole received empirical antifungal therapy (P = 0.19).

No serious adverse events related to treatment were reported by patients, and there was no treatment discontinuation because of drug-related adverse events in both groups.

The present analysis shows that micafungin did better than fluconazole in preventing invasive aspergillosis after transplant in these high-risk hematological diseases, as expected. In addition, micafungin was more effective than itraconazole in preventing all IFI episodes when also considering possible fungal infections. Future prospective studies would shed light on this issue, concerning this increasingly used transplant platform.

## Introduction

Invasive fungal infections (IFIs) such as Candida and Aspergillus species have caused significant morbidity and mortality among immunocompromised patients in the recent years. Infection is a primary cause of death in allogeneic hematopoietic stem cell transplant (allo-HSCT) recipients, with a fatality rate ranging from 50 to 80% after allo-HSCT.^1^–^3^ Recently, Girmenia et al., in a prospective study, reported a lower attributable mortality rate (AMR),^4^ possibly due to the improvement of diagnostic and therapeutic tools against infectious complications over the last years. The risk of developing IFIs is mainly related to the degree of immunosuppression, duration of neutropenia, the disruption of protective skin and mucosal surface barriers, graft vs. host disease (GVHD) and the use of corticosteroids.^5^–^7^ At the same time, haploidentical HSCT (haplo-HSCT) recipients are at high risk of IFI involving Candida and Aspergillus species.^2^,^8^

The introduction of post-transplant high-dose cyclophosphamide (PT-Cy) improved the feasibility of haplo-HSCT since an unmanipulated graft is infused without an elevated risk of GVHD.^9^,^10^ However, IFI prophylaxis, reported in several series, is disparate and reflects the policy of each center.^11^–^15^ As a consequence, the best antifungal prophylaxis in this type of transplant has yet to be determined.

In a recent study in the setting of haplo-HSCT with PT-Cy, we observed that the use of micafungin has a good safety and tolerability profile, with efficacy in preventing IFI in this high-risk population.^16^ We compare here the safety and efficacy of three distinct antifungal prophylaxis (micafungin, fluconazole or itraconazole) in patients undergoing haploidentical transplantation using T cell replete and PT-Cy in current practice.

## Patients and Methods

We conducted a retrospective comparative analysis in patients of two Cancer Center, who received itraconazole vs fluconazole or micafungin or for the prophylaxis of IFIs (Paoli-Calmettes Institute of Marseille in France) or Itraconazole vs fluconazole (Humanitas Cancer Center of Rozzano in Italy) while applying common transplant approaches and procedures during the study period. Eligible were adult patients who required a transplant from a haploidentical related donor for the treatment of various high-risk hematological malignancies. They had a history of disease relapse after several chemotherapeutic lines or after a previous autologous or allogeneic HSCT; or they were refractory to conventional salvage chemotherapy and thus, were candidates for an auto-allo strategy consisting of autologous HSCT followed by haplo-HSCT.^17^ Main patients’ and transplant characteristics are shown in Table 1. Patients with prior IFI were excluded. Study procedures were reviewed and approved by the institutional review board in both comprehensive cancer centers before patient enrollment started. Written informed consent was obtained from each patient before treatment.

### Conditioning and GVHD prophylaxis

Conditioning regimens consisted of association of: fludarabine, cyclophosphamide and total body irradiation (TBI) 2 Gy (n=80); thiotepa, fludarabine and busulfan (two or three days) (n=15); thiotepa, fludarabine and cyclophosphamide (n=4). As GVHD prophylaxis patients received PT-Cy at at a dose of 50 mg/kg i.v. on days +3 and +4; at day +5 cyclosporin A (3 mg/kg/day) or tacrolimus (1 mg/day) together with mycophenolate mofetil (15 mg/kg/day) were started. Filgrastim was prescribed at a dose of 5 mg/kg/day until neutrophil recovery.

### Infection prophylaxis and supportive care

The antifungal prophylaxis regimen was chosen according to institutional guidelines, regarding patients considered at high risk to develop IFI. Micafungin was used during a period due to the availability of this drug. After this period, fluconazole replaced micafungin. Itraconazole was administered mainly in the first part of haplo program. Micafungin was administered once daily as a 1-h infusion (50 mg/day), fluconazole orally at a dose of 400 mg/day, itraconazole 200mg/day intravenously. Patients received antifungal prophylaxis from the beginning of the conditioning regimen until hospital discharge, or the occurrence of an IFI when empirical or targeted treatment was started. The remaining antimicrobial prophylaxis consisted of acyclovir, sulfamethoxazole/trimethoprim, levofloxacin (this latter only at Humanitas Cancer Center). Supportive care, as well as diagnostic procedures of infections, was similar in the two centers.

### Efficacy and Safety Assessments

The incidence of proven or probable IFI in the three groups was calculated. Criteria for diagnosis were those for IFI as described by the Invasive Fungal Infections Cooperative Group of the European Organization for Research and Treatment of Cancer, National Institute of Allergy and Infectious Diseases Mycoses Study Group.^2^ Proven infection was defined as biopsy-proven invasive or disseminated infection. Sinus or pulmonary infection with Aspergillus, Fusarium, or Zygomycetes organisms also was considered to be proven if results of cultures of specimens obtained from the respiratory tract were positive in conjunction with compatible diagnostic imaging findings. Patients were deemed to have probable pulmonary aspergillosis if lower respiratory tract diagnostic studies revealed fungal elements in conjunction with compatible clinical and radiographic findings. Fungal infection was defined as suspected if fevers (temperature, > 38°C), persisted for > 96 h during the neutropenic phase, despite broad-spectrum antibacterial therapy, and led to the initiation of empirical antifungal therapy.^2^,^7^

Severe, non-hematological adverse event related to antifungal drugs, death from any cause, time to death, non-relapse mortality (NRM) and GVHD were evaluated as well. NRM and progression-free survival (PFS) were also estimated after 1 year of follow-up.

### Statistical analysis

Categorical variables were expressed as proportions and continuous variables as the median with the respective range. Comparisons were performed with the Chi-square and the Mann- Whitney test, or the Mood’s median test when appropriate, for categorical and continuous variables, respectively. Probabilities of overall survival (OS) were estimated by the Kaplan-Meier method, using the log-rank test for univariate comparisons; the cumulative incidence of IFI and NRM was calculated by a competing risk analysis, with the Gray test for univariate comparisons. Death was the competing event when calculating IFI, relapse or progression was competing event when calculating NRM. A p-value <0.05 was considered significant. Statistical analysis was performed with SPSS software (version 15.0) and R (version 2.12.2), package “cmprsk”.

## Results

From January 2009 to May 2013, a total of 99 patients were identified; 30 patients were treated with micafungin, and 69 patients were treated with itraconazole (n=50) or fluconazole (n=19). Main patients’ and transplant characteristics are detailed in Table 1. The most representative diagnoses are: Hodgkin’s lymphoma (HL), non-Hodgkin’s lymphoma (NHL) and acute myeloid leukemia (AML), accounting for 35%, 30% and 13% of patients’ population, respectively. The median follow-up from haplo-HSCT was 13 months (range: 5–23) in the micafungin group, 18 months (1–51) in the itraconazole and 3 months (1–6) in the fluconazole group (p<0.001).

### Engraftment and GVHD

Rate of engraftment was 89%. The median number of days to reach an ANC more than 500/mm 3 was 20 (14–42) days in the micafungin, 20 (6–32) in the itraconazole and 21 (15–38) in the fluconazole group (p=0.835). The median number of days to reach a platelet count more than 20 × 109/l was 29 (14–47) days in the micafungin, 27 (8–187) in the itraconazole and 30 (10–53) in the fluconazole group (p=0.497). The cumulative incidence of acute GVHD grade 2–4 was 22% (95% confidence interval [CI]: 9–40), 24% (95% CI: 12–38) and 26% (95% CI: 3–58) in the micafungin, itraconazole and fluconazole groups, respectively (p=0.951). The cumulative incidence of acute GVHD grade 3–4 was 12% (95% CI: 3–27), 2% (0–11) and 0% in the micafungin, itraconazole and fluconazole groups, respectively (p=0.163). The cumulative incidence of extensive chronic GVHD was 0%, 8% (95% CI: 1–22) and 0% in the micafungin, itraconazole and fluconazole groups, respectively (p=0.481).

### Invasive fungal infections

Proven or probable IFI were reported in 3 patients (10%) in the micafungin group, 5 patients (10%) in the itraconazole and 2 patients (11%) in the fluconazole group (p= 0.998). The cumulative incidence of proven/probable IFI was 11% (95% CI: 5–18). As expected, the rate of aspergillosis was higher after fluconazole prophylaxis compared with the micafungin and itraconazole groups (11% vs. 3% and 6%, respectively, p=0.589; see Table 2). Taking into account also possible IFI, aspergillosis occurred in 2 patients after fluconazole (11%) vs. two patients after micafungin (7%) vs. 13 patients after itraconazole (26%, p=0.148).

The etiology of the proven/probable IFI was Aspergillus (n=6), Candida albicans (n=2) and Candida non Albicans (n=2). The clinical syndrome was pneumonia in 7 cases, colitis in 1 case, sepsis in 1 case and sinusitis in 1 case. The median day of occurrence of proven/probable IFI was 28 days after haplo-HSCT (range: 0–69). In 7 out of 10 cases, the patient was experiencing neutropenia at the time of infection. No patient was diagnosed with acute GVHD before the onset of proven/probable IFI.

As reported in Figure 1A, the cumulative incidence of proven/probable IFI was 10% (95% CI: 2–24), 11% (95% CI: 4–22) and 12% (95% CI: 2–33) in the micafungin, itraconazole and fluconazole groups, respectively (p=0.998).

The cumulative incidence of proven/probable/possible IFI was 13% (95% CI: 4–28), 24% (95% CI: 13–38) and 12% (95% CI: 1–33) in the micafungin, itraconazole and fluconazole groups, respectively (p=0.402, see Figure 1B).

A total of 4 (13%) patients in the micafungin group vs 13 (26%) patients in the itraconazole group and 10 (53%) patients in the fluconazole received empirical antifungal therapy (P = 0.19). After hospital discharge, 18 patients (60%) of the micafungin group received fluconazole 400 mg/day until day 90 after haplo-HSCT because of the lack of GVHD and 17 patients (57%) received posaconazole because they presented acute GVHD. In the itraconazole group 14 patients (28%) presented acute GVHD received posaconazole after hospital discharge. Patients in the fluconazole group continue under fluconazole until day 90 after haplo-HSCT.

Invasive aspergillosis occurred in 1 (3%) patient while receiving micafungin, 3 (6%) while receiving Itraconazole and 2 (11%) patients while receiving fluconazole. Invasive candidiasis occurred in 2 (7%) patients while receiving micafungin, 2 (4%) patients while receiving itraconazole, and none while receiving fluconazole. (Table 2)

### Adverse events

No serious adverse events related to antifungal treatment were reported and there was no treatment discontinuation because of drug-related adverse event in any group. In the micafungin group, no infusion-related adverse event nor hepatotoxicity were observed. Elevated bilirubin levels of NCI-CTC grade 3 (or 4) reported in two patients were related to their haematological disease.

### Overall survival, progression-free survival, and non-relapse mortality

OS was 65% (95% CI: 46–83), 62% (95% CI: 47–76) and 95% (95% CI: 85–100) in the micafungin, itraconazole and fluconazole group, respectively (p=0.387). NRM at 100 days and 1 year was 7% (95% CI: 1–21), 17% (95% CI: 8–29), 0% and 12% (95% CI: 3–28), 24% (95% CI: 13–38), 0% in the micafungin, itraconazole and fluconazole groups, respectively (p=0.097). Causes of NRM were: pneumonia (n=1), JC virus encephalopathy (n=1), GVHD (n=1), graft failure (n=1) in the micafungin group and pneumonia (n=5), JC virus encephalopathy (n=1), septic shock (n=1), liver failure (n=1), heart failure (n=1), multiorgan failure (n=1), secondary cancer (n=1) in the itraconazole group. One death attributable to IFI was recorded in the itraconazole group and the etiologic agent was probable Aspergillosis.

A total of 13 (13%) patients died of disease relapse or progression after haplo-HSCT; diagnoses were: HL (n=3), NHL (n=3), AML (n=2), multiple myeloma (n=2), other (n=2). There was no significant difference in mortality due to relapse among the three groups (p=0.658).

## Discussion

We conducted a retrospective analysis to evaluate the safety and efficacy of micafungin itraconazole and fluconazole for the prophylaxis of IFI in a high-risk population of 99 consecutive patients undergoing haplo-HSCT and we found comparable IFI incidence among the three groups; as expected, fewer episodes of invasive aspergillosis occurred after micafungin and itraconazole vs. fluconazole, and this was particularly true in micafungin group where 12% of patients experienced grade 3–4 acute GVHD thus were at very high risk of developing IFI compared with the other 2 groups.

Haplo-HSCT represents an alternative option for patients without an otherwise suitable stem cell donor and the recent introduction of PT-Cy as part of GVHD prophylaxis permits the use of unmanipulated stem cells; nevertheless, infections after transplant (including IFIs) still represent a major cause of morbidity and mortality.^9^,^13^,^18^ Despite the availability of several antifungal molecules that allowed the reduction of mortality attributable to IFI in the last years, the optimal prophylaxis is still to be determined in patients before haplo-HSCT, although an anti-mold agent is recommended in such patients.^19^–^21^

Our findings confirmed the safety and efficacy of micafungin in preventing the occurrence of IFIs when administered to this specific population of high-risk patients. Although it is likely that patients’ outcome relies mainly on factors other than antifungal prophylaxis, the present results are in favour of the prophylactic use of micafungin in such a setting of patients, based on its safety profile and efficacy in preventing IFIs.^16^,^20^

We acknowledge several limitations in the present study: more patients in the micafungin group where managed in one center and it cannot be excluded that a difference in local epidemiology of fungal infection and environmental factors or indication for empirical treatment, could have influenced the rate of IFI under prophylaxis. Moreover, the relatively small number of patients in each group may have hidden some effect since we cannot exclude a lack of power in detecting a potential difference between the micafungin and the other two groups. However, the haplo-HSCT procedure, prophylactic strategy and supportive care were largely similar in both centers, as well as the patient characteristics, thus limiting the risk of bias due to local hospital care and patients selection. Only a randomized prospective trial may provide the answer to this relevant question.

In conclusion, this comparative study between three distinct antifungal prophylaxis for patients undergoing haplo-HSCT showed that micafungin was at least comparable than fluconazole in preventing invasive aspergillosis after transplant in these high-risk hematological diseases. Present findings contribute to defining a place for micafungin in prophylaxis after haplo-HSCT and provide additional information on this relevant issue, in an attempt to find the optimal prophylaxis strategy in this setting of high-risk patients.

## Figures and Tables

**Figure 1A:**
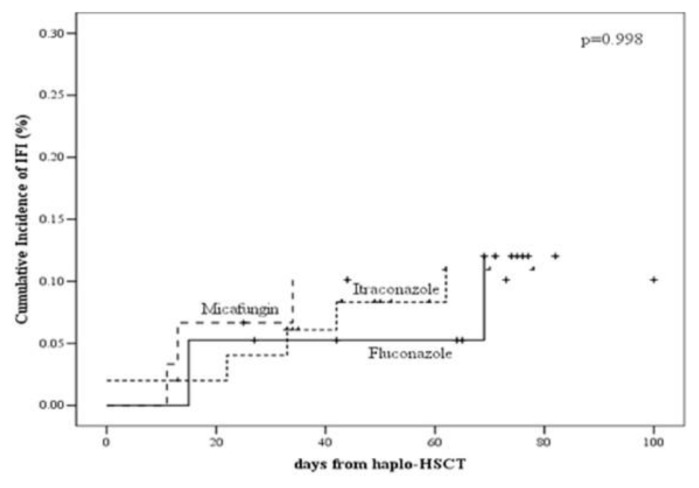
The cumulative incidence of proven/probable IFI in the micafungin, itraconazole and fluconazole groups.

**Figure 1B:**
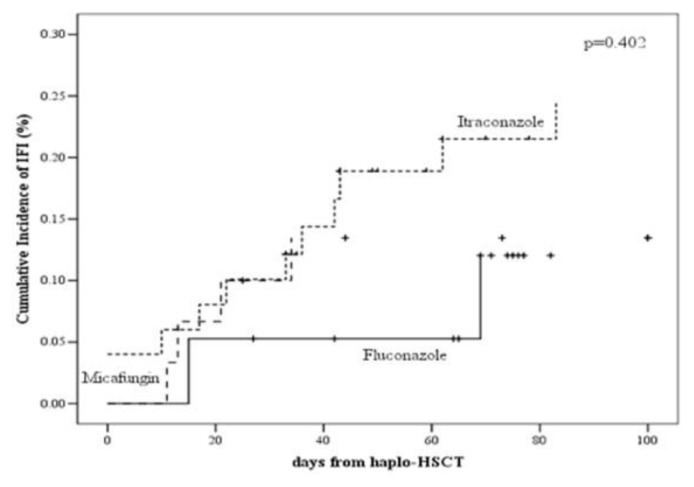
The cumulative incidence of proven/probable/possible IFI in the micafungin, itraconazole and fluconazole groups.

**Table 1:** Patients and transplantations characteristics.

Patients and transplant Characteristics Total =99	Micafungin n= 30 (30%)	Itraconazole n= 50 (51%)	Fluconazole n=19 (19%)	p-value

**Patient Age (median) [range]**	52 (20–64)	45 (19–68)	58 (30–65)	0.044

**Patient sex**				
Male	16 (53)	31 (62)	12 (63)	0.702
Female	14 (47)	19 (38)	7 (37)	

**Disease type**				
HL	8 (27)	25 (50)	2 (11)	
NHL	6 (20)	16 (32)	8 (42)	
AML	3 (10)	7 (14)	3 (16)	
ALL	3 (10)	-	-	0.198
MM	3 (10)	-	4 (21)	
CLL	4 (13)	2 (4)	-	
MDS	2 (7)	-	2 (11)	
MF	1 (3)	-	-	

**Status of disease at haplo-HSCT**				
CR	14 (47)	29 (58)	12 (63)	
PR/SD	10 (33)	14 (28)	5 (26)	0.466
PD	6 (20)	7 (14)	2 (11)	

**Previous auto-HSCT**	13 (43)	40 (80)	11 (65)	0.003
**Previous allo-HSCT**	6 (20)	3 (6)	1 (5)	0.098
**Tandem auto-allo-HSCT**	2 (7)	15 (30)	3 (16)	0.037

**Donor type**				
Sibling	19 (63)	23 (46)	6 (32)	
Parent	7 (23)	13 (26)	2 (11)	0.084
Child	4 (13)	13 (26)	11 (58)	
Other	-	1 (2)	-	

**Donor/recipient sex mismatch**	16 (53)	22 (44)	8 (42)	0.658

**ABO incompatibility**	5 (17)	9 (18)	9 (47)	0.067

**HCT-**				
**CI 0**	9 (30)	18 (36)	4 (21)	
**1–2**	4 (13)	16 (32)	4 (21)	0.042
**>2**	17 (57)	16 (32)	11 (58)	

**Conditioning regimen intensity**				
RIC	28 (93)	47 (94)	19 (100)	0.530
MAC	2 (7)	3 (6)	-	

**Stem cell source**				
Peripheral Blood	16 (53)	2 (4)	18 (95)	<0.001
Bone Marrow	14 (47)	48 (96)	1 (5)	

**Stem cell dose median [range]**				0.004
CD34+ × 10^6^/kg	4.1 (0.8–10.8)	3.6 (1.3–7.7)	5.0 (2.7–14.0)	

**Legend:** NHL, non-Hodgkin lymphoma; HL, Hodgkin lymphoma; AML, acute myeloid leukaemia; MM, multiple myeloma; MDS, myelodysplastic syndrome; MF, myelofibrosis; CLL, chronic lymphocytic leukaemia; CR, complete remission; PR, partial remission; PD, progressive disease; auto-HSCT, autologous hematopoietic stem cell transplant; allo-HSCT, allogeneic hematopoietic stem cell transplant; HCT-CI, hematopoietic cell transplant Comorbidity index; RIC, reduced intensity conditioning; MAC, myeloablative conditioning; Flu, fludarabine; Bu, busulfan; ATG, anti-thymocyte globulin; TT, Thiotepa; GVHD, graft versus host disease; Cy, Cyclophosphamide. NRM, non-relapse mortality.

When more than two categories were present, the statistical tests were performed comparing the following variables. Disease type: myeloid malignancies vs other; status of disease at haplo-HSCT: CR vs other; donor type: sibling vs other; HCT-CI: ≤2 vs >2.

**Table 2:** The incidence rates of Proven, Probable, or Possible Invasive Fungal Infections.

Infection at any time during the study	Micafungin n= 30	Itraconazole n= 50	Fluconazole n=19	*p value*

**Proven infection**				
Aspergillosis	-	-	-	
Candidiasis	2 (7%)	2 (4%)	-	0.513

**Probable infection**				
Aspergillosis	1 (3%)	3 (6%)	2 (11%)	0.589
Candidiasis	-	-	-	

**Possible infection**				
Aspergillosis	2 (7 %)	13 (26%)	2 (11%)	0.148
Candidiasis	-	-	-	
